# A Collision Risk Model to Predict Avian Fatalities at Wind Facilities: An Example Using Golden Eagles, *Aquila chrysaetos*

**DOI:** 10.1371/journal.pone.0130978

**Published:** 2015-07-02

**Authors:** Leslie New, Emily Bjerre, Brian Millsap, Mark C. Otto, Michael C. Runge

**Affiliations:** 1 U.S. Geological Survey, Patuxent Wildlife Research Center, Laurel, MD, United States of America; 2 Washington State University Vancouver, Vancouver, WA, United States of America; 3 U.S. Fish and Wildlife Service, Patuxent Wildlife Research Center, Laurel, MD, United States of America; 4 U.S. Fish and Wildlife Service, Division of Migratory Bird Management, Albuquerque, NM, United States of America; University of Lleida, SPAIN

## Abstract

Wind power is a major candidate in the search for clean, renewable energy. Beyond the technical and economic challenges of wind energy development are environmental issues that may restrict its growth. Avian fatalities due to collisions with rotating turbine blades are a leading concern and there is considerable uncertainty surrounding avian collision risk at wind facilities. This uncertainty is not reflected in many models currently used to predict the avian fatalities that would result from proposed wind developments. We introduce a method to predict fatalities at wind facilities, based on pre-construction monitoring. Our method can directly incorporate uncertainty into the estimates of avian fatalities and can be updated if information on the true number of fatalities becomes available from post-construction carcass monitoring. Our model considers only three parameters: hazardous footprint, bird exposure to turbines and collision probability. By using a Bayesian analytical framework we account for uncertainties in these values, which are then reflected in our predictions and can be reduced through subsequent data collection. The simplicity of our approach makes it accessible to ecologists concerned with the impact of wind development, as well as to managers, policy makers and industry interested in its implementation in real-world decision contexts. We demonstrate the utility of our method by predicting golden eagle (*Aquila chrysaetos*) fatalities at a wind installation in the United States. Using pre-construction data, we predicted 7.48 eagle fatalities year^-1^ (95% CI: (1.1, 19.81)). The U.S. Fish and Wildlife Service uses the 80^th^ quantile (11.0 eagle fatalities year^-1^) in their permitting process to ensure there is only a 20% chance a wind facility exceeds the authorized fatalities. Once data were available from two-years of post-construction monitoring, we updated the fatality estimate to 4.8 eagle fatalities year^-1^ (95% CI: (1.76, 9.4); 80^th^ quantile, 6.3). In this case, the increased precision in the fatality prediction lowered the level of authorized take, and thus lowered the required amount of compensatory mitigation.

## Introduction

Perceived as ‘green technology’, investment in wind energy has increased with the support of governments and individuals seeking to address climate change and reduce dependency on carbon-emitting fossil fuels [1–4]. However, wind facilities have the potential to negatively affect the environment [4–6]. Avian fatalities at operational wind facilities are of particular concern (e.g., [7–10]), and can be in conflict with regional laws (e.g., Migratory Bird Treaty Act (MBTA), 16 U.S.C. §§ 703–712; EC Directive on the Conservation of Wild Birds, 79/409/EEC).

High rates of avian fatalities do not occur at every wind facility (e.g., [11]), and the effect of an installation seemingly varies by species (e.g., [12]). The geographic location, local topographical features, species’ life history, and other factors all potentially play a role in the number of fatalities occurring [5], [13–15]. Yet even knowing these fatalities occur, the development of wind facilities has proceeded rapidly, outstripping the pace of study of their environmental effects [5]. As a result, uncertainty abounds regarding the relationships between wind facilities and avian mortality (e.g., [16]), making it difficult to predict the impact of an installation in advance of its construction.

The difficulty in prediction has led to a recognized need to better understand and anticipate the number of birds killed by collisions with operating turbines [17]. There are a number of different Collision Risk Models (hereafter referred to as CRM, e.g., [18–20]). Most existing methods of modeling predicted fatalities, however, do not incorporate uncertainty into their estimates and only provide point estimates from which to make management and conservation decisions. As a result, there is a false sense of confidence in their results [21].

To address this problem, we seek to predict, in advance, the number of fatalities at a proposed wind facility by developing an approach that explicitly incorporates and acknowledges uncertainty in the parameter estimates and predictions. We consider three components leading to avian fatalities at wind facilities: (1) the probability of collision, (2) the birds’ exposure and (3) a measure of the spatial and temporal extent over which a bird is at risk of collision (the hazardous footprint). While simpler than previous approaches, our method incorporates existing knowledge and avoids a number of biologically unrealistic assumptions about the constancy of bird flight height and speed [19]. To incorporate uncertainty into our model we use a Bayesian analytical framework, which allows us to define prior distributions for our model parameters that encompass best current biological knowledge and whose posterior distributions reflect the site-specific data collected pre-construction. The uncertainty quantified in these distributions is echoed in the pre-construction fatality predictions; these predictions can be updated and their uncertainty reduced if post-construction carcass monitoring data become available.

We provide a detailed description of our CRM for estimating avian fatalities and of our model-fitting approach. The aim of our work is to provide a method for estimating avian fatalities in the face of uncertainty. Our approach is designed to be accessible to the many ecologists, managers, policy makers and members of industry currently struggling with planning decisions for wind facilities. To illustrate its applicability to management and conservation we demonstrate our CRM using a golden eagle (*Aquila chrysaetos*) case study in the United States.

The effect of wind energy development on golden eagles is of particular concern because of high levels of mortality observed at some installations (e.g., [9]). Golden eagles are long-lived, with a low reproductive rate and delayed maturity [22]; consequently, a population’s viability is at risk when exposed to high levels of anthropogenic sources of mortality [23]. Furthermore, within the United States, government agencies such as the U.S. Fish and Wildlife Service (FWS) have a regulatory responsibility to manage and conserve golden eagles under the Bald and Golden Eagle Protection Act (BGEPA, 16 U.S.C. §§ 668–668d).

Under BGEPA, it is illegal to cause the death of a bald (*Haliaeetus leucocephalus*) or golden eagle unless in the possession of a FWS permit, even if the fatality occurs as an accident during an otherwise legal activity. To avoid a violation of BGEPA, a wind facility should obtain a permit prior to its construction. This requires a way to predict the number of eagle fatalities before they occur because each facility will only be allowed a set number of fatalities based on their anticipated risk to eagles. Our model makes this practicable, providing a framework for permitting decisions in which the explicit inclusion of uncertainty makes it possible to estimate the risk associated with the proposed wind installation. Because the FWS has also determined that golden eagle populations in the U.S. are unable to sustain any additional sources of anthropogenic mortality, wind facilities must offset any fatality they cause through compensatory mitigation. Our CRM’s predictions can also serve as an initial estimate of the level of mitigation required, allowing for pro-active conservation measures.

## Methods

### Model

The basis of our CRM is the assumption that there is a predictable relationship between pre-construction avian exposure (*λ*, bird-min hr^-1^ km^-3^) and subsequent fatalities (*F*, birds year^-1^) resulting from collisions with wind turbines. This relationship is dependent on a project’s hazardous footprint (*ε*, hr km^3^ year^-1^) and the species collision probability (*C*, birds bird-min^-1^). This gives [24]:

F=ελC. (1)

All collisions are assumed to remove a bird from the population.

**Fig 1:**
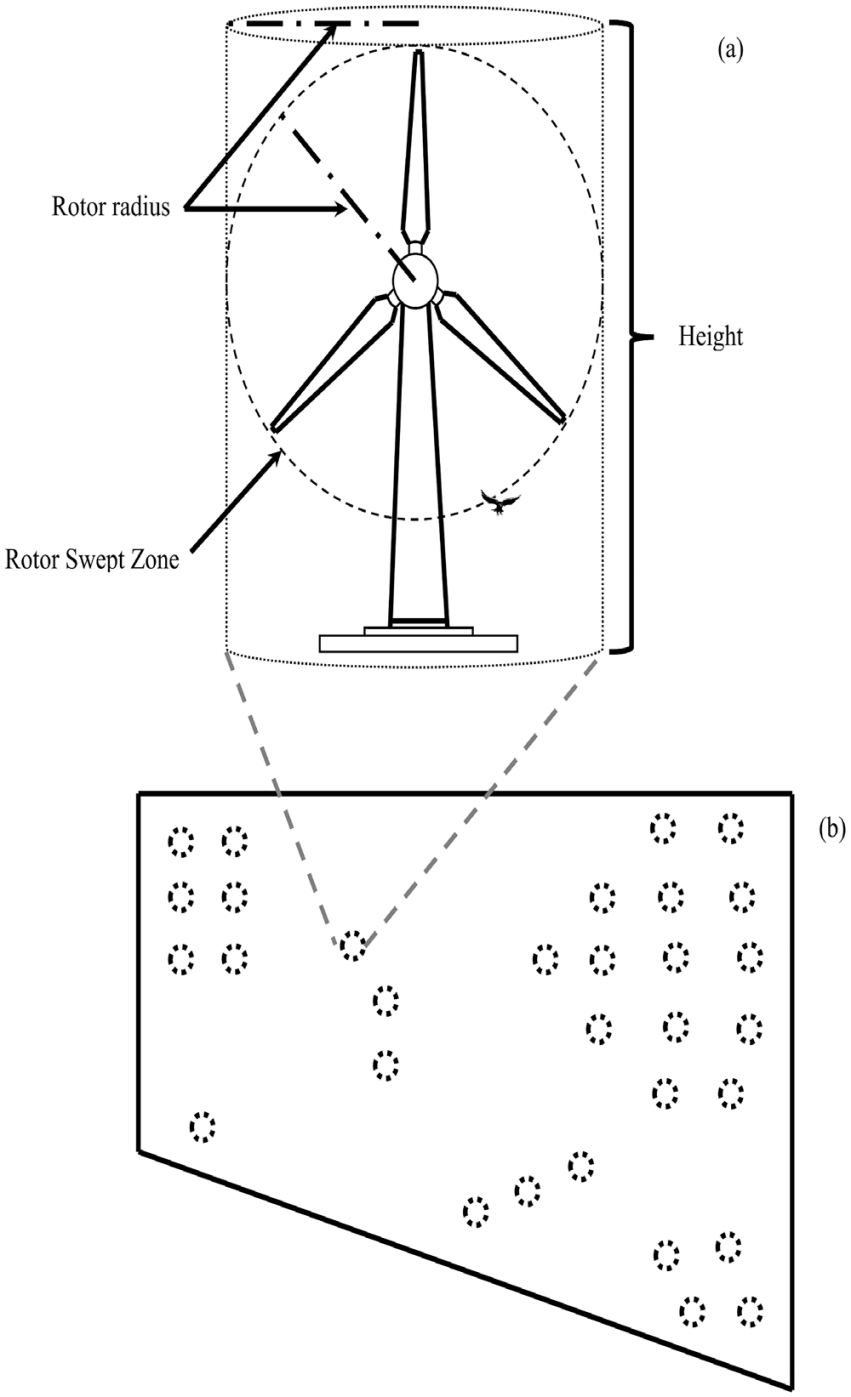
Diagram of a wind turbine and proposed project layout. The total hazardous volume (dotted line) for each turbine (a) is calculated using the rotor radius and the turbine height rather than just the rotor swept area. The total hazardous volume informs a wind facility’s hazardous footprint (circles) once the number of turbines within the project’s boundaries (solid line) is taken into account (b).

The parameter *C* incorporates the probability of a bird being hit by a turbine blade when within the turbine’s hazardous space and any change in exposure from pre- to post-construction. We define a turbine’s hazardous space as a cylinder with a defined height (e.g., 0.2 km, which approximately equals the height of the Enercon E-126, the tallest land-based wind turbine currently in production) whose radius extends from the center of the turbine’s hub to the tip of a rotor blade (Fig 1). This definition of *C* integrates the probability of collision over the entire space in which a bird is in proximity to a turbine and thus at greater risk. As a result, we combine the probability of collision at a non-varying height (i.e., constant height and speed) and the avoidance rate (including displacement from an area [19]) into one parameter, our collision probability, *C*. By extending the hazardous area surrounding each turbine from ground level to a defined height, we directly incorporate heterogeneity and any changes in bird flight behavior (e.g., [25], [26]) into *C*. In addition, we avoid data errors imposed by observers’ difficulty in accurately estimating flight height in the field [17], [19], [27]. When data are available, *C* can be estimated for individual wind facilities and can potentially be linked to covariates such as season or habitat.

Bird exposure (*λ* **)** is calculated as a function of the total survey effort [24], so that:

λ=k/ω, (2)

where *k* is the total number of bird minutes (rounded up to the nearest minute) counted across all surveys and *ω* (hr km^3^) is the effort in space and time put into collecting *k* (e.g., plot volume * count duration * number of plots for a point count survey). Different approaches may be taken to collect data on *λ* (e.g., point count, transects), provided that information on bird minutes and space and time surveyed are recorded. Regardless of the method of data collection, it should follow the tenets of good sampling design and be appropriate for the species under consideration. This includes ensuring the data are representative of the time and space under consideration and are unbiased. Eq 2 assumes *λ* is uniform across the space and time of the project footprint. If there is adequate spatial and temporal coverage, this assumption can be relaxed and *λ* can be stratified to account for factors such as migratory periods or differences in avian use due to habitat variation within the project footprint.

The last parameter, *ε*, is referred to as the expansion factor, because multiplying by *ε* expands the bird deaths hr^-1^ km^-3^ computed by the product of *λ* and *C* to the project’s hazardous footprint. The constant is calculated from the wind facility’s total relevant hours of operation (*τ*, hr), the height of the turbine’s hazardous space (*h*) and the area swept by a turbine’s rotor blades [24], giving:

$$\varepsilon =\tau nh\pi r^{2},$$ (3)

where *n* is the total number of turbines in a project, and *r* is the rotor radius. The value for *τ* will depend on the relevant periods of activity (e.g., diurnal, crepuscular, etc.) for the bird species being considered. The type of wind turbine may vary within a project. This can be accounted for by including separate *n* and *r* ^2^ terms for each turbine type, so that $\varepsilon =\tau \pi (n_{1}h_{1}r_{1}^{2}+n_{2}h_{2}r_{2}^{2}+\ldots +n_{T}h_{T}r_{T}^{2}),$ where 1,…,*T* denotes the turbine types used in the installation. We assume birds can only collide with actively rotating rotor blades. Therefore, *τ* should allow for periods when turbines may be shut down for maintenance, weather, or any committed curtailments.

### Model Fitting

We take a Bayesian approach to model fitting because of the relative ease with which it accounts for both uncertainty and prior information [28], [29], [30]. Bayesian inference draws statistical conclusions in terms of probability statements, conditioned on the observed data. Even when there is only partial knowledge, statements can be made about a system in a systematic, repeatable way that directly accounts for uncertainty [29]. The defined prior distributions are updated according to the data, resulting in posterior probability distributions from which all inferences are made [31]. The more data available, the less influence of the priors and the greater the reduction in uncertainty [32]. In addition, a Bayesian approach allows us to take advantage of a property called conjugacy, in which the posterior distribution maintains the same parametric form as its prior [29]. As a result, we can simulate directly from the posteriors [29] to obtain estimates of bird fatalities (*F*).

Bird minutes (*k*) are positive, whole numbered counts, allowing us to assume the data are distributed according to a Poisson distribution (e.g., [20]). From the conjugate family we chose a gamma distribution for the prior on *λ*, ensuring our estimate remains real-valued and positive and that the posterior on *λ* is also a gamma distribution. The parameter *C* is defined as the probability of a bird death per minute of pre-construction exposure, so each fatality is equivalent to a bird minute in which a bird collided with a turbine. This assumes no more than one fatality per bird-minute. Therefore, *F* arises from a binomial process where the probability, *p*, is equal to *C*, and the number of trials, *N*, is the total number of bird minutes in the wind facility’s hazardous footprint (*λε*). From the conjugate family we chose a beta distribution for the prior on *C*, ensuring our estimate is constrained to be between zero and one and that the posterior is also a beta distribution. Therefore, the posteriors for *λ* and *C* are,

$$\begin{array}{c} \lambda \sim \Gamma \left(\alpha +k,\beta +\omega \right)\\ C\sim \beta \left(\alpha \prime +F,\beta \prime +\lambda \varepsilon -F\right), \end{array}$$ (4)

where *α* and *β* and *α'* and *β'* are the parameter values for the gamma and beta priors, respectively. These parameters are species-specific.

### Priors

**Fig 2:**
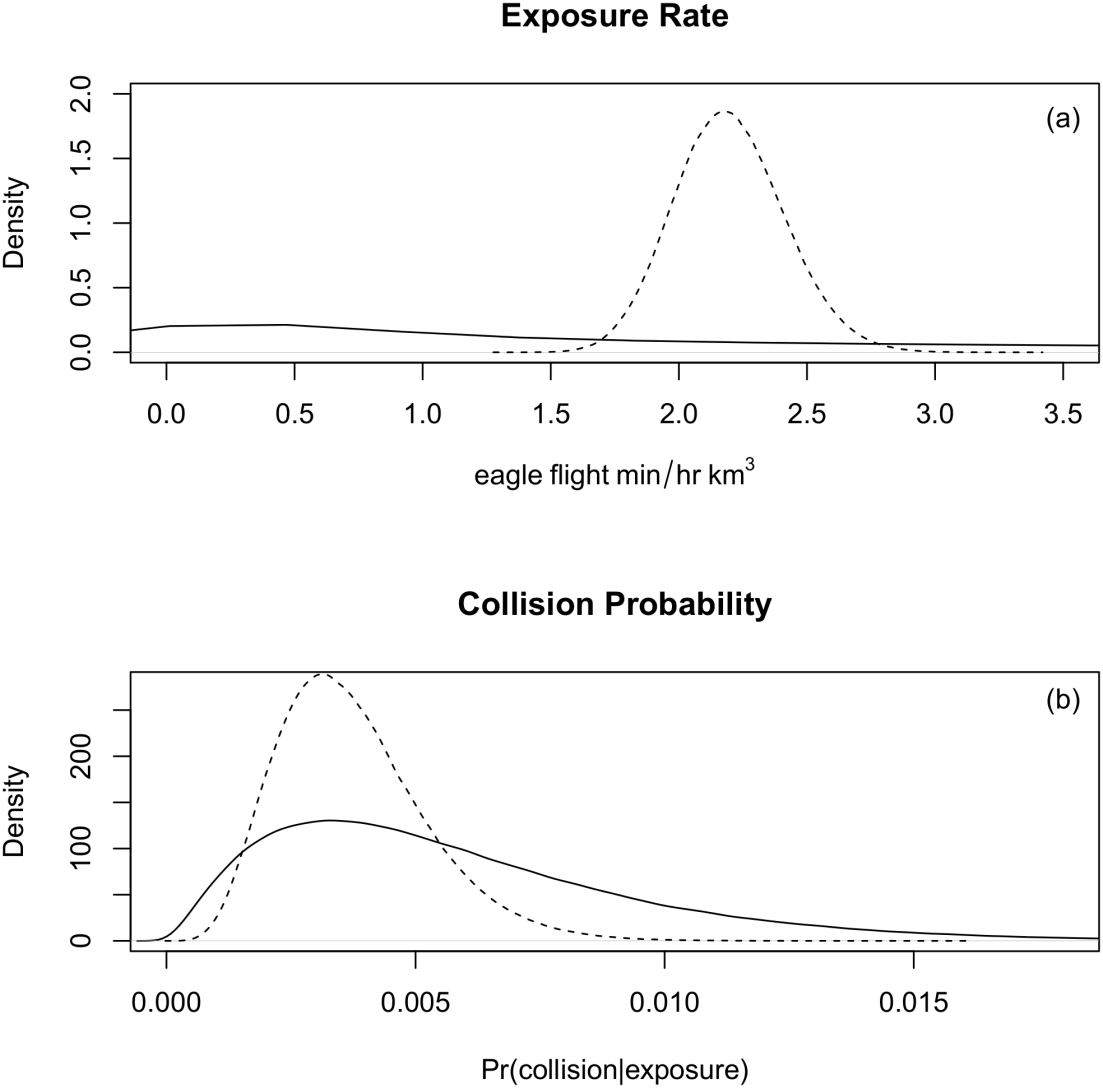
Posterior-prior plots for the golden eagle collision risk model. The prior (black line) and posterior (dashed line) distributions for the exposure rate (*λ*) (a) and collision probability (*C*) (b) of golden eagles at a wind facility in Wyoming. Both plots demonstrate how the inclusion of data results in a posterior distribution with reduced uncertainty that is more specific to the given wind facility.

It is necessary to define prior distributions for exposure (*λ*) and collision probability (*C*). These should be built using data from other locations that represents the best available biological knowledge and are representative of the site and species under consideration. In the absence of appropriate information, tools such as expert elicitation may be used (e.g., [33]). Alternatively, uninformative priors, which explicitly model the lack of knowledge, may also be used. For golden eagles we defined the species-specific prior for *λ* from known rates of eagle exposure (eagle-min km^-3^) on existing wind facilities (Table 1). The prior’s mean and variance were calculated from a mixture distribution based on the project-specific data, which were assumed to come from gamma distributions. This resulted in a Γ(0.415, 0.0472) prior (mean: 8.79 eagle-min hr^-1^ km^-3^, SD: 13.64) that represents our current knowledge on the range of possible eagle exposure rates (Fig 2a). For *C*, there are limited data relating exposure to eagle fatalities. To avoid an uniformed *β*(1,1) prior, which would result in a mean collision probability of 0.5, we used data available on golden eagle fatalities from four independent wind facilities in the U.S. (analyzed in [34]) to construct a *β*(2.31, 396.69) prior for *C* (mean: 0.0058 eagles eagle-min^-1^, SD: 0.0038, Fig 2b) [24].

**Table 1:** The data on golden eagle exposure used to construct the prior for *λ* (bird-min hr^-1^ km^-3^). Data on the mean and variance of *λ* were collected at nine independent sites within the U.S., all with varying levels of exposure. Site names are redacted in order to protect proprietary information.

Site	*λ*	*σ*
	(bird-min hr^-1^ km^-3^)	
A	16.1	4.64
B	0.375	0.375
C	4.90	2.19
D	44.4	6.62
E	0.672	0.154
F	4.67	0.503
G	1.26	0.194
H	3.01	0.549
I	3.79	0.493

### Golden eagle data

The data for our case study come from an existing wind facility in Converse County, Wyoming at which data were collected both before and after the installation’s construction. Prior to construction the facility defined 10 point count locations on the landscape. Each point was surveyed multiple times for 20 min duration, between 21 March 2008 and 1 November 2009. The total number of point counts performed, summed across all 10 locations, was 351. The point counts had a fixed radius of 0.8 km, and the number of minutes eagles were observed flying at or below 0.2 km was recorded. Therefore, we were able to calculate effort as,

$$\omega =n_{c}t_{c}h_{c}\pi r_{c}^{2},$$ (5)

where *n*_*c*_ is the total number of surveys, *t*_*c*_ is their duration in hours, *h*_*c*_ is the maximum height to which eagle minutes were recorded and *r*_*c*_ is the points’ fixed radius. As a result, *ω* = 47.05 hrs km^3^. Within this time and area a total of 103 eagle flight minutes were observed. Data on eagle minutes are collected from repeated surveys at the same locations, forming a longitudinal data set. However, averaging to a single datum (Eq 2) destroys any information within the sampling units. As a result, any dependence in the data due to the collection method is negated and pseudo-replication is not an issue [35–37].

The installation itself consists of 110 turbines, 66 with a 0.0385 km radius and 44 with a 0.0505 km radius, which are assumed to operate for 4460.147 annual daylight hours (*τ*). We used only daylight hours, defined as from sunrise to sunset, since golden eagles are a diurnal raptor [22]. The hazardous time should be specific to when a species is active and at risk (i.e., turbines are operating). As a result, where there is evidence that eagles may be active before dawn or after dusk, the hazardous time could be extended to include these periods. Given annual daylight hours, and using 0.2 km for the height of the hazardous space we calculated the facility’s hazardous footprint as *ε* = 588.62 hrs km^3^ (Eq 3).

Once the facility was operational, two years of fatality monitoring (i.e., carcass search surveys) were conducted from November 2010 until November 2012 at 36 turbines spread throughout the project. In the spring (16 March– 31 May) and autumn (1 August– 31 October) turbines were searched weekly, while in the summer (1 June– 30 July) and winter (1 November– 15 March) the turbines were searched bi-monthly. Search plots were 160 m square and centered on the turbine. Within the plot, the observers walked transects six to eight m apart while scanning the ground for fatalities.

In addition to the carcass monitoring, the facility performed carcass persistence trials (e.g., [38]), calculating an average daily persistence rate of 99.25% (SE: 10^−4^), to account for how long an eagle carcass would be available for detection. They also performed searcher efficiency trials (e.g., [39], [40]), resulting in an estimate of the observers being able to detect 85.67% (SE: 0.012) of carcasses on the landscape. Both parameters are key factors in estimating the true number of fatalities to have occurred (e.g. [39–41]). There are a variety of avian fatality estimators in the literature, and the choice of method will influence potential biases in the estimates (see [40–45] for more information). These estimators are distinct from CRMs because they are used to estimate the number of fatalities after they have occurred, rather than predict the number of fatalities before they have occurred. We used Péron et al.’s [40] avian fatality estimator and obtained an estimate of 7.96 (SE: 0.015) eagle fatalities over two years, or an averaged 3.98 fatalities year^-1^, for the facility.

### Golden eagle results

To predict the pre- and post-construction fatalities at the wind facility, we coded a Gibbs sampler based on Eqs 1 and 4 in the statistical programming language R [46]. A Gibbs sampler is the simplest algorithm that can be implemented to obtain samples from the posterior distribution via Markov chain Monte Carlo [29]. The code to implement the model with data collected at wind facilities both pre- and post-construction is available in the supplementary material (S1 simFatal Function).

Given the 103 observed eagle-minutes (*k*) and the values for *ω* and *ε*, we obtain a pre-construction mean posterior prediction of 7.48 eagle fatalities year^-1^ (95% CI: (1.1, 19.81)), based on an exposure rate of 2.2 eagle-min hr^-1^km^-3^ (SD: 0.22, Fig 2a) and the collision probability prior. For the FWS, the agency’s risk tolerance is expressed by using the 80^th^ quantile of the posterior distribution on *F* in the permitting process [24], which would be equal to 11.01 eagle fatalities year^-1^ for the wind facility.

Using the same code (S1 simFatal Function), but including information on the estimated number of true fatalities from the carcass monitoring surveys reduces the post-construction mean posterior fatality prediction to 4.8 eagle fatalities year^-1^ (95% CI: (1.76, 9.4)) and the collision probability to 0.0037 eagles eagle-min^-1^ (SD: 0.0015, Fig 2b), while the posterior estimate of *λ* is unchanged. Given the FWS’ risk tolerance, the 80^th^ quantile for the updated fatality prediction would be 6.34 eagle fatalities year^-1^.

## Discussion

We have constructed a straightforward collision risk model that directly incorporates uncertainty and can readily be adapted as information becomes available. We assumed a Poisson distribution for bird flight minutes and a binomial distribution for the number of fatalities. We do this in order to ensure conjugacy, so that ecologists, managers, policy makers and industry that are not familiar with a Bayesian analytical approach do not need to worry about issues such as convergence or mixing (see [29] for more information) when assessing and making inferences from the model outputs. However, different, non-conjugate distributions can be readily specified if deemed biologically appropriate. This would necessitate the use of a different Markov chain Monte Carlo algorithm, such as a Metropolis-Hastings, to obtain the posterior distributions of interest [29] and thus would require more statistical skill on behalf of the user.

As with existing CRMs (e.g., [19]), our model assumes the bird population is open, which is biologically realistic at the scale of an individual project. However, in most other ways the CRM presented here differs from other models currently in the literature (e.g., [18–20]). Existing CRMs differentiate between a collision probability based on non-varying avian flight (i.e., a straight line at a constant height and speed) and an avoidance rate incorporating a bird’s ability to evade a collision (e.g., [19]). Because of the assumption of non-varying flight, existing models only consider the area swept by a turbine’s rotor blades to be hazardous (e.g., [18], [19]), since a bird flying above or below the rotor blades is presumed to be unable to change its trajectory. As well as being biologically unrealistic, this requires accurate measures of bird flight height, which are difficult to obtain in the field [17], [19], [27] and necessitates that there be no change in turbine specifications between planning and construction. In contrast, our model directly incorporates heterogeneity into *C*, avoids a known source of uncertainty and gives greater flexibility to the wind facility operators. There are new methods to model avian flight height that include uncertainty [47], which may improve this aspect of existing CRMs once it is fully incorporated into the models.

Another advantage to our CRM is the direct inclusion of uncertainty around the predictions of avian fatalities and parameter estimates, making it possible to directly assess the risk associated with different development scenarios. Furthermore, as data targeted to reduce known uncertainties (e.g., carcass monitoring) are collected, the estimates of *C* and predictions of annual avian fatalities will improve. This can facilitate reassessment of decisions and conservation actions in an adaptive management framework [48–51]. Where existing studies have failed to account for post-construction data in future environmental-decision making [52], our approach is designed to take advantage of new knowledge, consequently adjusting the model and management decisions.

In our golden eagle example, we demonstrated how the CRM can be applied to obtain a fatality prediction, with uncertainty, prior to a wind facility’s construction. By using the 80^th^ quantile of the pre-construction posterior distribution on *F*, the FWS takes a risk-averse approach to eagle conservation with the initial permit decision. This is because if the pre-construction prediction approximates the truth, then 80% of the time the actual number of fatalities will be less than the number for which the wind facility is permitted, thus supporting the species’ conservation. This approach is also risk-averse for the wind facility operator because it means there is only a 20% chance that they will exceed the fatalities authorized by the permit.

Once a facility is operational, carcass monitoring protocols in conjunction with an unbiased fatality estimator (e.g., [40]) make it possible to assess whether the facility has met its permit conditions. The FWS can use the new prediction of future golden eagle fatalities to adjust the facility’s permit, accounting for the new, site-specific information. Whether the new prediction of eagle fatalities uses the mean or 80^th^ quantile is a policy decision that may be influenced by auxiliary information, such as eagle density over the monitored time period or the perceived risk to the raptors (e.g., [53]). Given the use of the 80^th^ quantile for the initial prediction, it is likely that the predicted number of fatalities will be adjusted downwards, so the wind facility may have initially mitigated for more golden eagles than were actually killed at the installation. The facility can claim credit for those eagles as operations continue, thus reducing the amount of compensatory mitigation required. Furthermore, the pro-active nature of the conservation measures ensures the facility will receive credit both for those efforts as well as the contributions those eagles make to the population, thus increasing the value of their initial investment in conservation.

Our model for predicting avian fatalities at wind facilities is designed to incorporate and account for uncertainty. This uncertainty is important to managers and permitting agencies as they evaluate risk. Further, the model framework provides a specific method for reducing this uncertainty, both within and across facilities, as new data become available, thus allowing for adaptive management. While simpler than other existing tools for prediction of avian fatalities at wind facilities, this model has minimal data requirements and provides guidance even in the absence of site-specific data. These qualities make our approach accessible to ecologists, managers, policy makers and industry for use in real world decision contexts.

## Supporting Information

S1 simFatal Function An R function for the implementation of the collision risk model. The same function can be used with data collected at wind facilities both pre- and post-construction.
(DOCX)
