# Supplementary material for: A Collision Risk Model to Predict Avian Fatalities at Wind Facilities: An Example Using Golden Eagles, Aquila chrysaetos
Source: PLoS One. 2015 Jul 2;10(7):e0130978. doi: 10.1371/journal.pone.0130978 (PMC4489750; doi:10.1371/journal.pone.0130978)
Supplement: S1 simFatal Function — The same function can be used with data collected at wind facilities both pre- and post-construction. (DOCX) [file pone.0130978.s001.docx]

**S1: simFatal function**

simFatal <- function(BMin=-1, Fatal=-1, SmpHrKm, ExpFac, aPriExp=1,

bPriExp=1,aPriCPr=1, bPriCPr=1){

# BMin: observed number of bird minutes

# Fatal: annual avian fatalities on an operational wind facility

# SmpHrKm: total time and area surveyed for bird minutes

# ExpFac: expansion factor

# aPriExp: alpha parameter for the prior on lambda

# bPriExp: beta parameter for the prior on lambda

# aPriCPr: alpha parameter for the prior on C

# bPriCPr: beta parameter for the prior on C

# The default of a negative value for BMin or Fatal indicates that no data were collected for those model inputs

require(rv)

# Update the exposure prior

if(BMin>=0){

aPostExp <- aPriExp + BMin

bPostExp <- bPriExp + SmpHrKm

}else{

aPostExp <- aPriExp

bPostExp <- bPriExp}

Exp <- rvgamma(n=1, aPostExp, bPostExp)

# Update the collisions prior

if(Fatal>=0){

aPostCPr <- aPriCPr + Fatal

bPostCPr <- ((rvmean(Exp) * ExpFac) - Fatal) + bPriCPr

}else{

aPostCPr <- aPriCPr

bPostCPr <- bPriCPr}

CPr <- rvbeta(n=1, aPostCPr, bPostCPr)

Fatalities <- ExpFac * Exp * CPr

attr(Fatalities,"Exp") <- c(Mean=rvmean(Exp), SD=rvsd(Exp))

attr(Fatalities,"CPr") <- c(Mean=rvmean(CPr), SD=rvsd(CPr))

return(Fatalities)}
